# The Frequency and Risk Factors of Acute Kidney Injury in Children with Oncological Diseases: A Single-Center Study in Bulgaria

**DOI:** 10.3390/children12050540

**Published:** 2025-04-23

**Authors:** Petya Markova, Antoniya Yaneva, Stoyan Markov, Mariya Spasova, Neofit Spasov

**Affiliations:** 1 Department of Pediatrics, Medical Faculty, Medical University of Plovdiv, 4000 Plovdiv, Bulgaria; petya.markova@mu-plovdiv.bg (P.M.); mariya.spasova@mu-plovdiv.bg (M.S.); neofit.spasov@mu-plovdiv.bg (N.S.); 2 Department of Pediatrics, University Hospital “St. George”, 4000 Plovdiv, Bulgaria; 3 Medical Informatics, Biostatistics and eLearning, Medical University of Plovdiv, 4000 Plovdiv, Bulgaria; antoniya.yaneva@mu-plovdiv.bg; 4 Department of Otorhinolaryngology, Medical Faculty, Medical University of Plovdiv, 4000 Plovdiv, Bulgaria; 5 Department of Otorhinolaryngology, University Hospital “St. George”, 4000 Plovdiv, Bulgaria

**Keywords:** childhood oncological diseases, acute kidney injury in children

## Abstract

**Background:** Progress in the treatment of childhood oncological diseases has led to the prolonged survival of patients with this severe diagnosis. On the other hand, the prolonged chemotherapy courses that achieve this outcome also bring a number of complications, with acute kidney injury being one of them. Its occurrence in patients not only affects their quality of life but also prolongs and increases the cost of hospitalization, burdens the body with additional treatment, and impacts the ability to manage the underlying disease. **Aim:** The aim of this study is to determine the frequency of acute kidney injury among children hospitalized in the Pediatric Oncohematology Unit in Plovdiv during the period 2016–2020, as well as to identify the risk factors for its occurrence, its severity, and its dependence on tumor type, gender, and age. **Patients and Methods:** During the five-year period under review, a total of 213 newly diagnosed children with hematological diseases were admitted to our Pediatric Oncohematology Unit—122 boys and 91 girls. **Results:** Acute kidney injury was identified in 94 (44.1%) of the children—54 with solid tumors and 40 with malignant hemopathies. The main cause of acute kidney injury diagnosed was drug-induced nephrotoxicity, especially due to nephrotoxic chemotherapeutic agents. No statistically significant association was found between the type of tumor and the occurrence of acute kidney injury. Of the children with documented episodes of AKI, 11 were found to have CKD according to the KDIGO criteria. **Conclusions:** Acute kidney injury is a common complication that occurs during the medical treatment of children with malignant diseases.

## 1. Introduction

In recent decades, there has been considerable progress in improving the event-free survival of children with oncological diseases. This is due to advancements in chemotherapy concepts and protocols, the refinement of risk stratification, and the improvement of supportive care. The increasing number of childhood cancer survivors has brought to the forefront several important issues, such as reducing early and long-term treatment side effects and ensuring a good quality of life when planning long-term therapeutic strategies. Acute kidney injury (AKI) is one of the complications that occur in these patients during treatment and leads to prolonged hospitalization, increased treatment costs, higher mortality, and a reduction in functional capacity [1,2,3]. Various risk factors for its occurrence are discussed in the literature—drug-induced nephrotoxicity, the presence of tumor lysis syndrome, initial renal tumor infiltration, and septic complications [4,5]. The occurrence of AKI during treatment often requires changes to the therapeutic plan—dose modifications, the inclusion of alternative, less nephrotoxic medications from the same group, or even the exclusion of this cytostatic group from subsequent treatment. In the long term, the risk of progression to chronic kidney disease (CKD) remains significant. The information in the literature regarding the frequency of AKI in patients with oncological diseases primarily concerns adults. Data on the pediatric population are limited and highly variable (with a reported frequency ranging from 11% to 80%), depending on the method used for AKI staging and the patient cohort (the inclusion or exclusion of patients after bone marrow transplantation) [6,7,8,9,10,11,12,13].

## 2. Aim

The aim of this study is to determine the frequency of AKI among children hospitalized in the Pediatric Oncohematology Unit in Plovdiv during the period 2016–2020, as well as to identify the risk factors for its occurrence, its severity, and its dependence on tumor type, gender, and age.

## 3. Patients and Methods

A retrospective study was conducted over a five-year period (2016–2020) on children with oncological diseases who were treated in the Pediatric Oncohematology Department of the Pediatrics Clinic at UMHAT “St. George”, Plovdiv. The following criteria were applied:

Inclusion Criteria:

1. Age between 0 and 18 years;
2. The diagnosis of the primary disease—solid tumors or malignant hemopathies;
3. Newly diagnosed patients admitted for initial treatment.

Exclusion Criteria:

1. Children with pre-existing kidney diseases;
2. Children with CKD;
3. Patients who have undergone bone marrow transplantation.

The data were obtained from the patients’ electronic records. The analysis includes the following variables related to the occurrence of an acute kidney injury episode:

1. Age at the diagnosis of the oncological disease;
2. The type of disease (solid tumor or malignant hemopathy);
3. Renal infiltration at disease onset;
4. The presence of other comorbidities (heart failure, neurological impairment, liver dysfunction, respiratory failure, infection—including sepsis);
5. Evidence of acute tumor lysis syndrome;
6. Laboratory data at admission and during treatment (serum creatinine, urea, electrolytes, blood gas analysis, and urinalysis);
7. Chemotherapy with nephrotoxic agents (high-dose Methotrexate, Ifosfamide, Cisplatin, and Carboplatin).

All these parameters were used to assess the occurrence of AKI, determine its cause, and stage the disease. Events that occurred within 14 days before the onset of AKI were considered risk factors for its development. The severity of AKI was also analyzed.

## 4. AKI Criteria

The occurrence of an AKI episode was determined using the KDIGO criteria for AKI [14]:

Stage 1: An increase in serum creatinine by 26 µmol/L within 48 h or an increase in serum creatinine to up to 1.9 times the baseline level.

Stage 2: An increase in serum creatinine by 1.9–2.9 times the baseline level within a period of 7 days.

Stage 3: Serum creatinine increases by 3 times above the baseline value, or creatinine > 353.6 µmol/L, or the initiation of renal replacement therapy, or eGFR < 35 mL/min/1.73 m^2^ (<18 years old).

The second main criterion, oliguria, was not applied due to the lack of proper documentation of this parameter [14]. The occurrence of acute tumor lysis syndrome was determined using the Cairo–Bishop criteria [15,16,17,18].

The onset of CKD was determined based on the KDIGO 2022 criteria [19].

## 5. Statistical Methods

Data analysis for the study was performed using the statistical software SPSS v.23. Descriptive statistics were used to describe both quantitative and qualitative variables. Categorical variables and grouped data are presented through frequency distributions and relative proportions. To assess the relationship between categorical variables χ2 criterion was used. A significance level of *p* < 0.05 was considered for the null hypothesis.

## 6. Results

### 6.1. The Characteristics of the Patients

The study included 213 newly diagnosed children over a five-year period, who were admitted for the first time for treatment in the pediatric oncohematology department. Patients who had already undergone treatment in other medical institutions were excluded from the study. The patients were divided into subgroups based on the following criteria.

**Figure 1:**
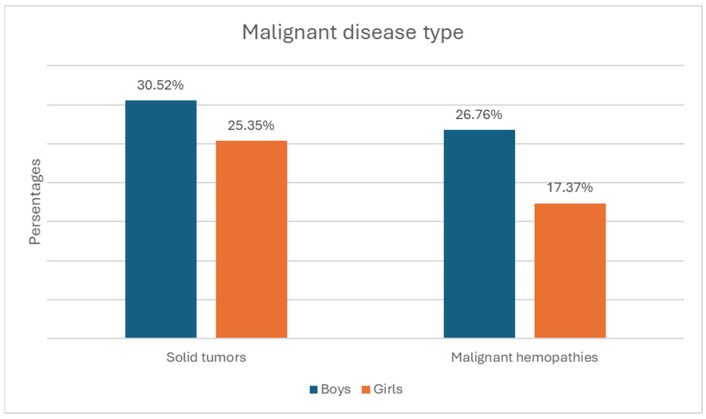
Distribution by gender and type of malignant disease.

**Figure 2:**
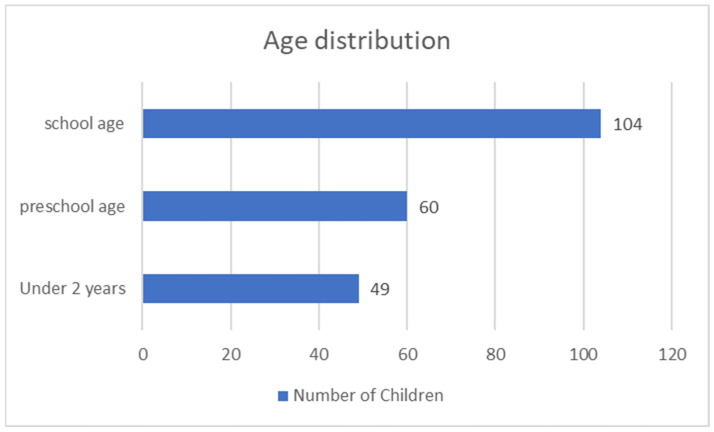
Distribution of children by age.

**Figure 3:**
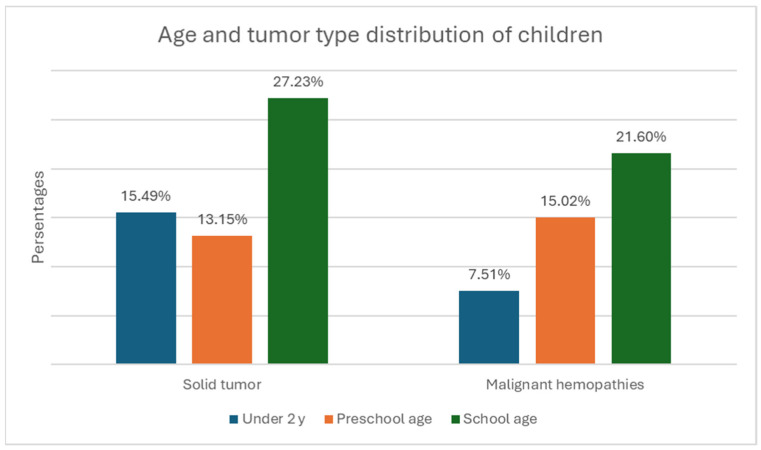
Distribution of children by tumor type and age at diagnosis.

1. Gender distribution—122 boys (57.3%) and 91 girls (42.7%). The relative proportion of boys compared to girls is significantly higher in both the solid tumor group and the malignant hematologic disorder group (Figure 1).
2. The age of onset divides the children into three subgroups—under 2 years, preschool age, and school age. In the first group, there were 49 children, (23% of all children); in the second, there were 60 children (28.2%); and in the third, there were 104 children (48.8%) (Figure 2). The group with the highest relative proportion are the school-age children, both in terms of solid tumors (27.23%) and malignant hematologic disorders (21.60%). However, there is no statistically significant difference in the distribution of age groups between the two types of malignant diseases (χ^2^ = 4.68, *p* = 0.096).
3. Malignant disease type—119 children (55.9%) with solid tumors and 94 (44.1%) with malignant hematologic disorders. The relative proportion of boys compared to girls is higher in both the solid tumor group and the malignant hematologic disorder group (Figure 3). However, this difference is not statistically significant (χ^2^ = 0.55, *p* = 0.458).

The kidney function of the patients was monitored throughout the entire course of treatment.

### 6.2. Acute Kidney Injury

**Figure 4:**
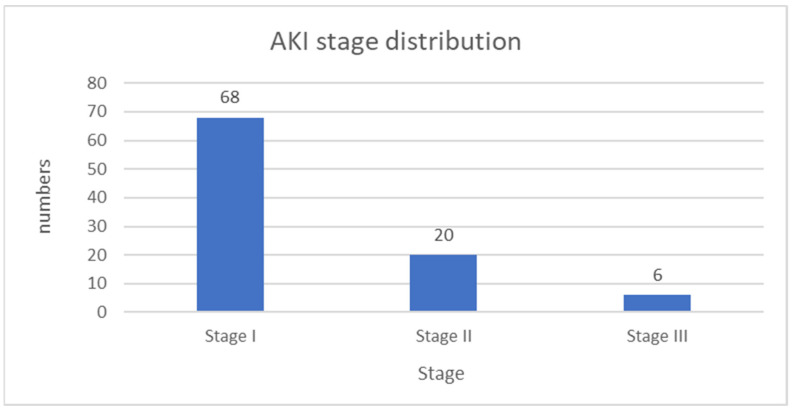
AKI stage distribution among children treated with nephrotoxic medications.

Using the KDIGO criteria, 94 children (44.1% of all children) were found to have experienced at least one episode of AKI, with 18 (19.15%) of them having more than one episode during their treatment. The highest percentage of cases were classified as stage 1 AKI—68 episodes, accounting for 72.3% of all AKI episodes. Stage 2 AKI was observed in 20 episodes (21.3%), while only 6 episodes (6.4%) were classified as stage 3 AKI according to KDIGO. The distribution of AKI severity is illustrated in Figure 4.

### 6.3. Acute Kidney Injury (AKI) Risk Factors

**Figure 5:**
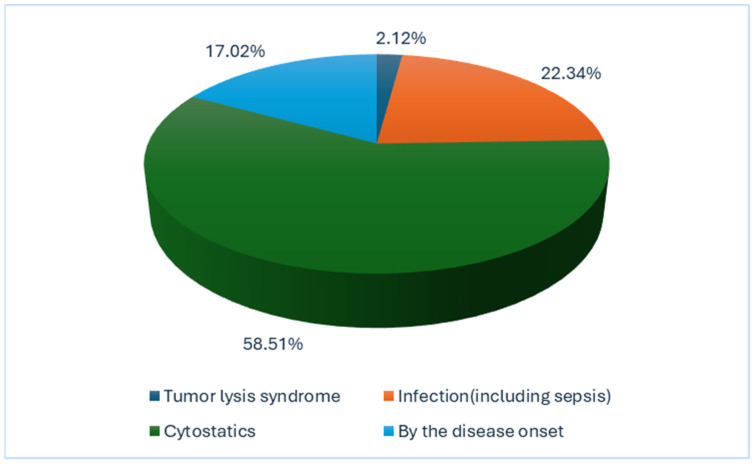
The frequency distribution of the AKI causes.

Regarding the etiology of AKI, the main contributing factors were drug-induced nephrotoxicity, particularly chemotherapy, infections—including septic infections during drug-induced aplasia—tumor lysis syndrome, and renal infiltration at disease onset. The highest relative share was attributed to drug-induced nephrotoxicity, specifically chemotherapy, accounting for 58.51% of all AKI episodes—see Figure 5.

**Figure 6:**
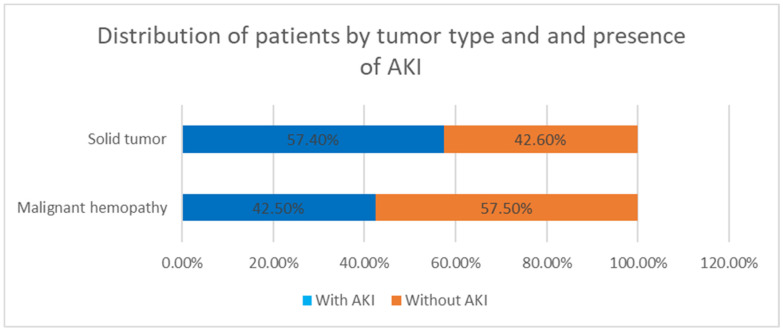
Distribution of patients by tumor type and presence of AKI.

Of the 94 children diagnosed with AKI, 54 (57.4%) had solid tumors, while 40 (42.5%) had malignant hemopathies (Figure 6). No statistically significant association was found between tumor type and the presence of AKI (χ2 = 0.170, *p* = 0.781, N = 213), nor between gender and the presence of AKI (χ2 = 0.105, *p* = 0.781, N = 213).

**Figure 7:**
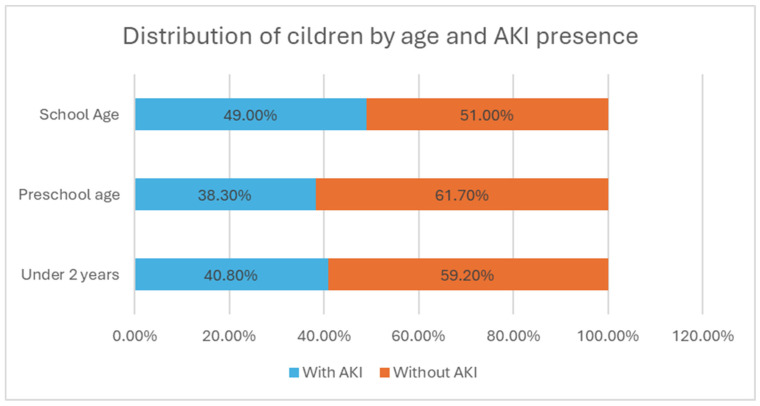
Distribution of children by age and AKI presence.

The highest incidence of AKI was observed among school-aged children (49%), see Figure 7. However, this difference was not statistically significant (χ2 = 2.05, *p* > 0.05).

Eighteen children experienced more than one episode of AKI, but no statistically significant association was found between the number of AKI episodes, tumor type (*p* = 0.401), and the cause of AKI (*p* = 0.787). The data are presented in Table 1.

**Figure 8:**
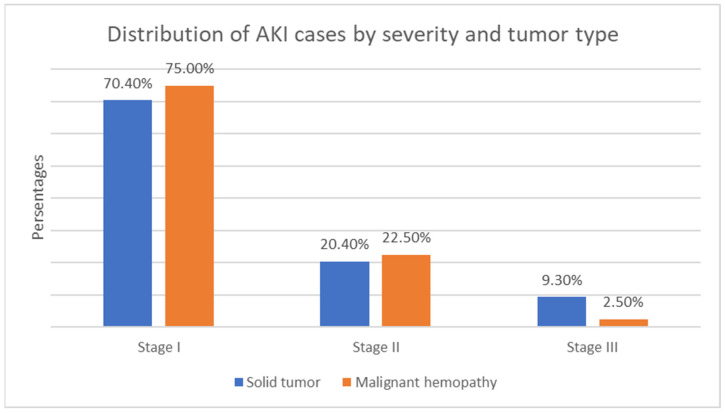
Distribution of AKI cases by severity and tumor type.

In both types of malignant diseases, Stage 1 AKI severity predominates, while Stage 3 severity, including the need for renal replacement therapy, was mostly observed in children with solid tumors (Figure 8).

**Figure 9:**
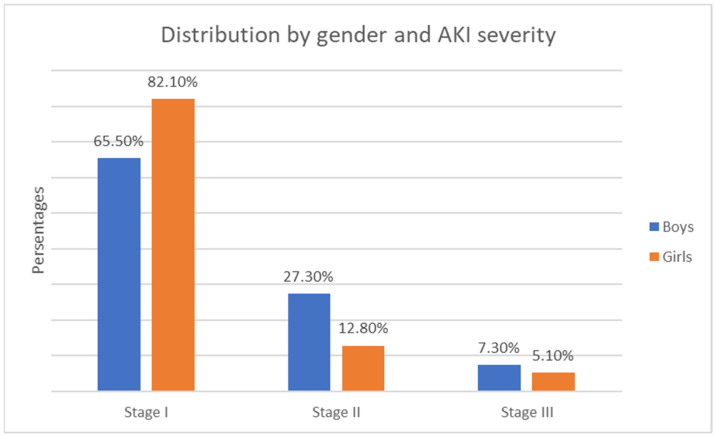
Distribution by gender and AKI severity.

Regarding gender, Stage 1 AKI severity was the most prevalent in both groups. No statistically significant difference was observed between gender and AKI severity (χ2 = 4.89, *p* = 0.087)—see Figure 9.

**Figure 10:**
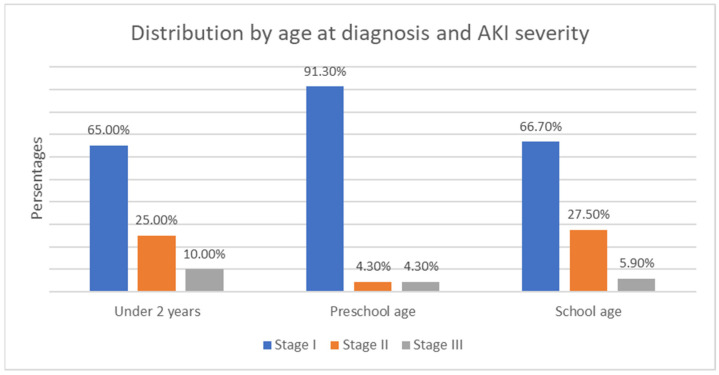
Distribution by age at diagnosis and AKI severity.

Regarding the age of onset, a higher frequency of AKI diagnosis and severity was observed among school-aged children and those under 2 years old. However, this difference was not statistically significant (χ2 = 7.25, *p* = 0.122)—see Figure 10.

**Figure 11:**
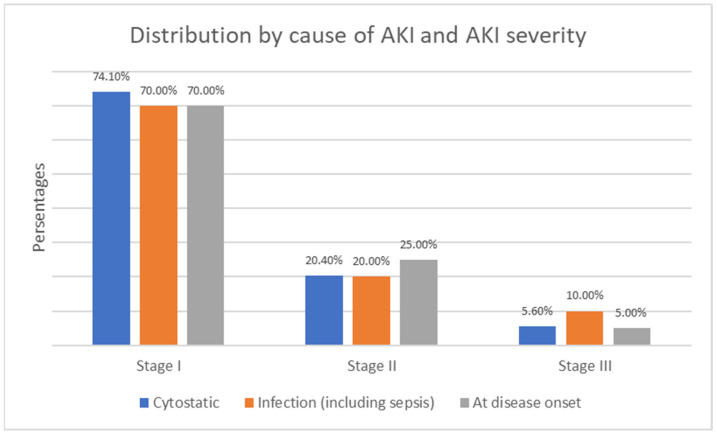
Distribution by cause of AKI and AKI severity.

The more severe Stage 2 and Stage 3 AKI cases were predominant at disease onset, including cases with tumor lysis syndrome and severe infections during aplasia. The most common cause of AKI—drug-induced nephrotoxicity—primarily led to Stage 1 AKI—see Figure 11.

The most nephrotoxic drugs are listed in Table 2.

Of the children with documented episodes of AKI, 11 were found to have CKD according to KDIGO criteria. Among them, eight children were G1A2 stage, two children G1A3, and one child G2A3 stage.

## 7. Discussion

The conducted retrospective study reveals a significantly high frequency of acute kidney injury (AKI)—44.1% among children with malignant diseases undergoing chemotherapy. This rate is similar to the frequency reported in the literature [1,2,3,4,5,6,7,8]. Table 3 presents data from literature studies on the incidence and etiology of AKI in pediatric oncology.

Park et al. studied 1868 children diagnosed with malignant diseases between 2004 and 2013 and found AKI in 983 of them (52.6%) during the first year of primary disease treatment. The highest frequency of AKI was reported in patients with acute lymphoblastic leukemia, followed by those with acute myeloblastic leukemia and medulloblastoma [4]. Mengqi Xiong et al. conducted a study between 2013 and 2015 on 9828 children with malignant diseases, where AKI was observed in 1657 patients (16.9%) [1]. The severity of AKI in these two studies was classified as stage 3 in approximately 50% of cases, requiring renal replacement therapy. In contrast, our results show a predominance of stage 1 AKI (72.3%), with only six children (6.4%) experiencing stage 3 AKI.

This discrepancy is likely explained by the primary mechanism of drug-induced nephrotoxicity in our group, reflected in AKI development. Causing primarily tubular damage, this type of injury does not lead to a significant increase in serum creatinine levels, which is a mandatory criterion in the severity grading scale of AKI. In contrast to the data published in the literature, this study shows a slight predominance of acute kidney injury (AKI) in children with solid tumors (57.4%) compared to those with malignant hemopathies (42.6%).

The development of AKI in children with oncological diseases is multifactorial. The presented study identified the following risk factors: drug-induced nephrotoxicity due to chemotherapy, infection (including septic infection), acute tumor lysis syndrome, and renal infiltration at disease onset. Similar risk factors have also been reported in the literature [1,4,6,22,23]. A significant number of studies highlight acute tumor lysis syndrome as a leading factor in the development of AKI [1,4,13]. In contrast to the literature data, our study identifies drug-induced nephrotoxicity, particularly chemotherapy, as the primary factor. This explains the higher incidence of AKI among patients with solid tumors, in whom nephrotoxic agents such as Ifosfamide, Methotrexate, Cisplatin, and Carboplatin are used. A probable explanation for the predominance of drug-induced nephrotoxicity in the present study is that each newly diagnosed patient was monitored for kidney function until the end of their treatment. Additionally, some patients experienced a relapse over time, requiring the reintroduction of therapy with nephrotoxic agents. In the cited studies, patients were monitored only during the first year of treatment. An additional factor is that most patients were not specifically examined for tubular damage, which is the primary pathophysiological mechanism of drug-induced nephrotoxicity. This likely led to the underdiagnosis of subclinical tubulopathy. If the therapy with the respective medications continues, the condition progresses to clinically apparent AKI. Regarding the age of onset of malignant diseases and the corresponding AKI episodes, the highest percentage of patients are of school age. Our study did not find a statistically significant association between the number of episodes, the severity of AKI, the type of malignant disease, and the patient’s gender. This is also confirmed by most studies in the literature.

A limitation of this study is its single-center design, which does not allow for statistically reliable conclusions regarding the overall incidence of AKI in children with oncological diseases undergoing chemotherapy across Bulgaria.

## 8. Conclusions

AKI is a common complication during the treatment of children with malignant diseases, with the incidence observed in our center reaching 44.1%—a rate comparable to data reported in the literature. Given the leading cause identified in our study—namely, chemotherapy-induced nephrotoxicity—there is a clear need to introduce additional biomarkers that are sensitive to the onset of tubular injury when monitoring these patients. All of this necessitates careful monitoring of kidney function in these children during treatment, the use of preventive measures when possible, and post-treatment surveillance to detect the onset of CKD [23,24].

## Figures and Tables

**Table 1:** Average number of AKI episodes according to AKI severity, etiology, and tumor type.

Category		Average Number of Episodes	Standard Deviation	Minimum	Maximum
**AKI severity**	Stage I	1.15	0.3964	1	3
	Stage II	1.4	0.5982	1	3
	Stage III	1.33	0.5164	1	2
**AKI cause**	Drug cytostatic	1.22	0.4196	1	2
	Infection; sepsis	1.3	0.6569	1	3
	Renal infiltration at disease onset	1.13	0.3416	1	2
**Tumor type**	Solid tumor	1.24	0.4733	1	3
	Malignant hemopathy	1.18	0.4465	1	3

**Table 2:** The most nephrotoxic drugs.

Neprotoxic Drug	Number of Children Who Developed AKI
Methotrexate	20
Ifosfamide	9
Cisplatin	2
Carboplatin	3

**Table 3:** Studies on the incidence of AKI among children treated for malignant diseases.

Year	Number of Patients	Disease Type	AKI Frequency
Park at all [4] 2004–2013	1868	Malignant hemopathies and solid tumors	52.6%
Xiong M. at all [1] 2013–2015	9829	Malignant hemopathies and solid tumors	16.9%
Plessis L. at all [20] 2018	53	Acute myeloid leukemia	64%
Tariq R. at all [21] 2017–2019	399	Malignant hemopathies and solid tumors	21.33%

## Data Availability

The original contributions presented in the study are included in the article, further inquiries can be directed to the corresponding author.
